# The effect of lipocortin 1 on neutrophil deformability

**DOI:** 10.1155/S0962935196000257

**Published:** 1996-06

**Authors:** E. M. Drost, W. MacNee, S. F. Smith

**Affiliations:** 1 Department of Medicine Rayne Laboratory University of Edinburgh Medical School, Teviot Place Edinburgh EH8 9AG UK; 2 Department of Medicine Charing Cross and Westminster Medical School Fulham Palace Road London W6 8RF UK

## Abstract

Lipocortn 1 (Lc1) is an anti-inflammatory protein, which, given systemically, inhibits polymorphonuclear neutrophil (PMN) emigration from the circulation to sites of inflammation; delivery of Lc1 to the inflamed site is ineffective. We have examined the effect of Lc1 on changes in PMN deformability, and observed a consistent improvement in the deformability of unstimulated PMN; N-formyl-methionyl-leucyl-phenylalanine (fMLP)-activated cell deformability was unaltered. A Lc1-induced increase in cell deformability may reduce PMN sequestration so contributing to the anti-migratory effects of systemic Lc1 previously demonstrated *in vivo*.


Mediators of Inflammation, 5, 188–190 (1996)

### The effect of lipocortin 1 on neutrophil deformability

E. M. Drost,[1,CA] W. MacNee[1] and S. F. Smith[2]

[1]Department of Medicine, Rayne Laboratory, University of Edinburgh, Medical School, Teviot Place, Edinburgh EH8 9AG, UK; and [2]Department of Medicine, Charing Cross and Westminster Medical School, Fulham Palace Road, London W6 8RF, UK

[CA]Corresponding Author

**LIPOCORTIN 1 (Lc1) is an anti-inflammatory protein, which, given systemically, inhibits polymorphonuclear neutrophil (PMN) emigration from the circulation to sites of inflammation; delivery of Lc1 to the inflamed site is ineffective. We have examined the effect of Lc1 on changes in PMN deformability, and observed a consistent improvement in the deformability of unstimulated PMN; *N*-formyl-methionyl-leucyl-phenylalanine (fMLP)-activated cell deformability was unaltered. A Lc1-induced increase in cell deformability may reduce PMN sequestration so contributing to the anti-migratory effects of systemic Lc1 previously demonstrated *in vivo*.**

**Key words:** Deformability, Lipocortin 1, Polymorphonuclear neutrophil

### Introduction

Glucocorticoids are powerful anti-inflammatory drugs capable of modulating both cellular and soluble components of the inflammatory response. In this investigation we have studied an anti-inflammatory, glucocorticoid-inducible protein, lipocortin 1 (Lc1, annexin 1), which mediates a number of glucocorticoid actions including inhibition of PMN emigration to sites of inflammation.[1] The mechanism of this action has not been fully elucidated, but could include effects on PMN rolling adhesion, expression of adhesion molecules, or diapedesis, since, to reach a site of inflammation, circulating PMN must slow down, adhere to the endothelium and migrate between the endothelial cells.

Most previous studies suggest that glucocorticoids and Lc1 do not directly alter expression of PMN or endothelial cell adhesion molecules,[2,3] an exception being Cronstein and colleagues[4] who demonstrated that glucocorticoids diminish ELAM-1 and ICAM-1 expression on LPS-stimulated endothelium,[4] although these changes appear to be both time and stimulus-dependent.[3,4]

Although the details of extravasation are thought to vary between the systemic and pulmonary circulations, a reduction in cell deformability can prolong the transit time of PMN through the microvasculature,[5] thus increasing opportunities for selectin-mediated rolling adhesion of PMN to endothelial cells, this being a prerequisite under shear conditions for the leucocyte firm (CD11/CD18-mediated) adhesion which is required for PMN emigration.[6] Therefore, the aim of the present study was to investigate the effect of Lc1 on PMN deformability.

### Materials and Methods

Recombinant human Lc1 (rhLc1) was the generous gift of Dr J. L Browning, Biogen Inc., Cambridge, MA. PMN populations ($n = 9$; $>96.9 \pm 2.2\%$ pure) were harvested from peripheral venous blood as described previously.[7]

Cell suspensions were pre-incubated with rhLc1 dissolved in PBS containing 1 mg human serum albumin/ml for 30 min at 37°C at a concentration of 2 μg/$10^6$ cells/ml after which the cells remained >98% viable. Lc1 binds to human peripheral blood leukocytes in a dose-dependent manner; the cell surface binding sites on PMN are approximately 80% saturated at the concentration of Lc1 and the temperature used in the present study.[8] Biologically inactive rhLc1 was used as a control.

Cell deformability was assessed in the presence and absence of $10^{-7}$ M *N*-formyl-methionyl-leucyl-phenylalanine (fMLP), by filtration of PMN suspensions ($1 \times 10^5$/ml PBS containing 5% albumin) at constant flow through a Nucleopore polycarbonate membrane (5 μm diameter pores).[9] The more rigid the cells, the longer they take to enter and pass through the pores resulting in a higher pressure. Hence, the pressure developed by a cell suspension over 6 min filtration compared to the pressure of PBS alone was measured as an indicator of cell deformability.

*Statistics:* Differences between treatments were assessed using a one-way analysis of variance

© 1996 Rapid Science Publishers

(ANOVA). The level of significance was $p < 0.05$.

### Results

Following pre-incubation with functional rhLc1, passive PMN from every subject ($n = 9$) showed increased deformability compared to control cells at each time point examined up to 6 min filtration (Fig. 1). However, no change in PMN deformability was observed in the presence of biologically inactive rhLc1 (6 min pressure; control, 3.09 ± 0.68 cm $H_2O$; inactive rhLc1, 3.00 ± 0.24 cm $H_2O$; $n = 5$; mean ± S.E.M.; $p > 0.05$ ANOVA).

When PMN were stimulated with fMLP (6 min presure; fMLP, 8.03 ± 1.33 cm $H_2O$) their deformability was significantly reduced compared to unstimulated cells (6 min pressure; control, 3.09 ± 0.68 cm $H_2O$). Neither pre-incubation with functional nor inactive rhLc1 had a consistent or significant effect on PMN deformability in the presence of fMLP (6 min pressure; fMLP + rhLc1, 8.49 ± 1.0 cm $H_2O$; fMLP + inactive rhLc1, 10.12 ± 0.7 cm $H_2O$; $n = 5$; $p > 0.05$ ANOVA).

Examination of PMN by light microscopy confirmed that no cell aggregation occurred with any treatment regimen, and thus the pressures generated were indeed a reflection of cell deformability.

### Discussion

In this study we observed that incubation of passive PMN with rhLc1 *in vitro* resulted in a consistent increase in cell deformability, which occurred only when the protein was in its correct, three-dimensional conformation. The dimensions of the microvasculature impose a restraint on PMN passage, particularly in the pulmonary capillaries.[7,10] The disparity between the diameters of circulating PMN (6–8 μm) and the systemic (6 μm) or pulmonary (5 μm) capillaries require that the cells must deform during capillary transit (reviewed in Reference 10). Thus, the more deformable the cell, the more rapid the transit through the capillary beds, the briefer the contact with the endothelium and the fewer the opportunities for the PMN to adhere prior to migrating out of the circulation. Hence, a Lc1-induced increase in cell deformability may decrease sequestration of PMN in the capillary bed; this mechanism could contribute to the reduction by intravenous Lc1 of experimentally induced neutropenia *in vivo*.[11]

Interestingly, when PMN were activated with fMLP, Lc1 had no effect on cell deformability.

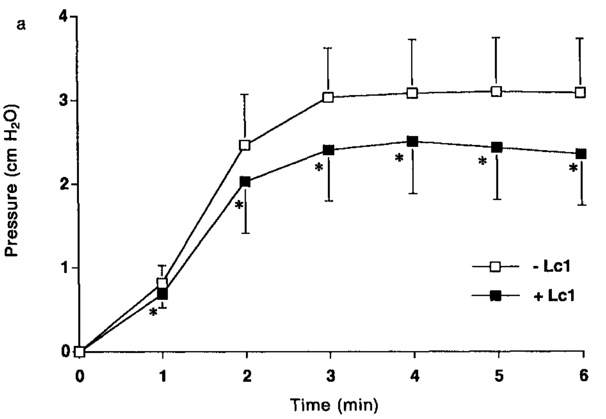

FIG. 1. Effect of rhLc1 on deformability of passive human PMN isolated from peripheral blood ($n = 9$). rhLc1 (2 μg/ml) significantly increased the deformability of passive PMN at each of the time points examined. *untreated cells $p < 0.05$ (ANOVA).

This is consistent with observations *in vivo* which have shown that whilst systemic Lc1 reduces the migration of (passive) circulating neutrophils, Lc1 administered directly to a site of inflammation has no effect on (activated) migrated PMN.[11]

How Lc1 causes the observed change in the mechanical properties of PMN is currently unknown. PMN deformability is determined mainly by the intracellular levels, conformation and distribution of filamentous (F) actin. Lc1 can bind F-actin[12] and thus might directly modify the conformation and/or distribution of this cytoskeletal protein. However, the effect of Lc1 on PMN deformability may potentially be mediated by changes in signal transduction since studies on A549 cells demonstrate that Lc1 can inhibit G-protein-mediated activation of arachidonic acid release.[13] However, as Lc1 treatment did not affect the deformability of activated PMN in our study, this mechanism seems less likely to explain the observation reported here. We plan to investigate the effect of Lc1 on F-actin in PMN more fully.

### References

1. Perretti M, Flower NJ. Modulation of IL-1-induced neutrophil migration by dexamethasone and lipocortin 1. *J Immunol* 1993; **150**: 952–999.
2. Mancuso F, Flower RJ, Perretti M. Leucocyte transmigration, but not rolling or adhesion, is selectively inhibited by dexamethasone in the hamster post-capillary venule. *J Immunol* 1995; **155**: 377–386.
3. Forsyth KD, Talbot V. Role of glucocorticoids in neutrophil and endothelial adhesion molecule expression and function. *Mediators Inflam* 1992; **1**: 101–106.
4. Cronstein BN, Kimmel SC, Levin RI, Matiniuk F, Weissmann G. A mechanism for the anti-inflammatory effects of corticosteroids: the glucocorticoid receptor regulates leucocyte adhesion to endothelial cells and expression of endothelial-leucocyte adhesion molecule 1 and intercellular adhesion molecule 1. *Proc Natl Acad Sci USA* 1992; **89**: 9991–9995.
5. Downey GP, Doherty DE, Schwab R, Elson EL, Henson PM, Worthen GS. Retention of leucocytes in capillaries: a role of cell size and deformability. *J Appl Physiol* 1990; **69**: 1767–1778.
6. Ley K, Lundgren E, Berger E, Arfors K-E. Shear-dependent inhibition of granulocyte adhesion to cultured endothelium by dextran sulfate. *Blood* 1989; **73**: 1324–1330.
7. MacNee W, Wiggs B, Belzberg A, Hogg JC. The effect of cigarette smoking on neutrophil kinetics in human lungs. *N Engl J Med* 1989; **321**: 924–928.
8. Goulding NJ, Luying P, Guyre PM. Characteristics of lipocortin-1 binding to the surface of human peripheral blood leukocytes. *Biochem Soc Trans* 1990: **18**: 1237–1238.
9. Lennie SE, Lowe GDO, Barbenel JC. Filterability of white blood cell subpopulations, separated by an improved method. *Clin Haemorheol* 1987; **7**: 811–816.
10. Hogg JC. Neutrophil kinetics and lung injury. *Physiol Rev* 1987; **67**: 1249–1295.
11. Peretti M, Ahlwalia A, Harris JG, Goulding NJ, Flower RJ. Lipocortin-1 fragments inhibit neutrophil accumulation and neutrophil-dependent oedema in the mouse. *J Immunol* 1993; **151**: 4306–4314.
12. Glenney JR, Tack B, Powell MA. Calpactins: two distinct $Ca^{++}$-regulated phospholipid- and actin-binding proteins isolated from lung and placenta. *J Cell Biol* 1987; **104**: 503–511.
13. Croxtall JD, Choudhury QAM, Tokumoto H, Flower RJ. Lipocortin-1 and the control of arachidonic acid release in cell signalling. *Biochem Pharmacol* 1995; **50**: 465–474.

ACKNOWLEDGEMENTS. We thank Dr T. Tetley for helpful comments on the manuscript. A Wellcome Travel Grant made to S.F.S. facilitated the writing up of the study.

**Received 12 December 1995; accepted 22 February 1996**

## References

[PDF_00203] Cronstein B. N., Kimmel S. C., Levin R. I., Martiniuk F., Weissmann G. (1992). A mechanism for the antiinflammatory effects of corticosteroids: the glucocorticoid receptor regulates leukocyte adhesion to endothelial cells and expression of endothelial-leukocyte adhesion molecule 1 and intercellular adhesion molecule 1.. Proc Natl Acad Sci U S A.

[PDF_00233] Croxtall J. D., Choudhury Q., Tokumoto H., Flower R. J. (1995). Lipocortin-1 and the control of arachidonic acid release in cell signalling. Glucocorticoids (changed from glucorticoids) inhibit G protein-dependent activation of cPLA2 activity.. Biochem Pharmacol.

[PDF_00230] Glenney J. R., Tack B., Powell M. A. (1987). Calpactins: two distinct Ca++-regulated phospholipid- and actin-binding proteins isolated from lung and placenta.. J Cell Biol.

[PDF_00219] Goulding N. J., Luying P., Guyre P. M. (1990). Characteristics of lipocortin 1 binding to the surface of human peripheral blood leucocytes.. Biochem Soc Trans.

[PDF_00225] Hogg J. C. (1987). Neutrophil kinetics and lung injury.. Physiol Rev.

[PDF_00208] Ley K., Lundgren E., Berger E., Arfors K. E. (1989). Shear-dependent inhibition of granulocyte adhesion to cultured endothelium by dextran sulfate.. Blood.

[PDF_00214] MacNee W., Wiggs B., Belzberg A. S., Hogg J. C. (1989). The effect of cigarette smoking on neutrophil kinetics in human lungs.. N Engl J Med.

